# A Small Compound KJ-28d Enhances the Sensitivity of Non-Small Cell Lung Cancer to Radio- and Chemotherapy

**DOI:** 10.3390/ijms20236026

**Published:** 2019-11-29

**Authors:** Hwani Ryu, Hyo Jeong Kim, Jie-Young Song, Sang-Gu Hwang, Jae-Sung Kim, Joon Kim, Thi Hong Nhung Bui, Hyun-Kyung Choi, Jiyeon Ahn

**Affiliations:** 1 Division of Radiation Biomedical Research, Korea Institute of Radiological & Medical Sciences (KIRAMS), Seoul 01812, Korea; hwanya85@kirams.re.kr (H.R.); hjkim@kirams.re.kr (H.J.K.); immu@kirams.re.kr (J.-Y.S.); sgh63@kirams.re.kr (S.-G.H.); jaesung@kirams.re.kr (J.-S.K.); 2 Department of Biology, Korea University, Seoul 02841, Korea; joonkim@korea.ac.kr; 3 Department of Medicinal Chemistry, Jungwon University, Goesan 28024, Korea; buihongnhung103@gmail.com

**Keywords:** poly (ADP-ribose) polymerase inhibitor, non-small cell lung cancer, DNA damage, radiotherapy, chemotherapy, combination therapy

## Abstract

We previously reported on a poly (ADP-ribose) polymerase (PARP) 1/2 inhibitor *N*-(3-(hydroxycarbamoyl)phenyl)carboxamide (designated KJ-28d), which increased the death of human ovarian cancer *BRCA1*-deficient SNU-251 cells. In the present study, we further investigated the antitumor activities of KJ-28d in *BRCA*-proficient non-small cell lung cancer (NSCLC) cells to expand the use of PARP inhibitors. KJ-28d significantly inhibited the growth of NSCLC cells in vitro and *in vivo*, and induced DNA damage and reactive oxygen species in A549 and H1299 cells. Combined treatment with KJ-28d and ionizing radiation led to increased DNA damage responses in A549 and H1299 cells compared to KJ-28d or ionizing radiation alone, resulting in apoptotic cell death. Moreover, the combination of KJ-28d plus a DNA-damaging therapeutic agent (carboplatin, cisplatin, paclitaxel, or doxorubicin) synergistically inhibited cell proliferation, compared to either drug alone. Taken together, the findings demonstrate the potential of KJ-28d as an effective anti-cancer therapeutic agent for *BRCA*-deficient and -proficient cancer cells. KJ-28d might have potential as an adjuvant when used in combination with radiotherapy or DNA-damaging agents, pending further investigations.

## 1. Introduction

Lung cancer is one of the most commonly occurring cancers worldwide. Non-small cell lung cancer (NSCLC) accounts for more than 84% of all lung cancers [1]. Despite recent advances in our understanding of the molecular and genetic basis of lung cancer and improvements in therapy, the 5-year survival rate of patients with NSCLC in both Korea (23.7%) and the United States (23%) is lower than that for many other leading cancers [1,2]. The standard chemotherapy treatment used for NSCLC is either the platinum-based (cisplatin, carboplatin, etc.) or taxane-based (paclitaxel, docetaxel, etc.) regimens, or epidermal growth factor receptor (EGFR) inhibitors. However, because of the lack of response or resistance to those therapies, many patients with NSCLC consider new alternative therapies or receive combination therapies [3]. Therefore, the development of novel drugs or strategies of combination therapy with existing drugs is urgently required.

Ionizing radiation (IR) is directly cytotoxic through the induction of DNA single- or double-strand breaks (DSB) and indirectly cytotoxic through the generation of reactive oxygen species (ROS), leading to tumor cell death and thus cancer treatment. Similarly, platinum- or taxane-based chemotherapeutic agents inhibit cell mitosis through the induction of DNA damage. DNA lesions induced by IR or cytotoxic agents can be repaired by six major DNA repair pathways: base excision repair, nucleotide excision repair, direct repair (i.e., O^6^-alkylguanine DNA alkyltransferase directly repairs DNA damaged lesions by removing O^6^-methylguanine), mismatch repair, homologous recombination (HR), and non-homologous end-joining pathways. NSCLC is characterized by genomic instability with mutations and translocations in oncogenes, such as the *Ki-ras2 Kirsten rat sarcoma viral oncogene homolog proto-oncogene* (*KRAS*), *EGFR*, *ALK receptor tyrosine kinase* (*ALK*), *ataxia-telangiectasia mutated* (*ATM*) or *phosphatidylinositol-4,5-bisphosphate 3-kinase catalytic subunit alpha* (*PIK3CA*), and tumor suppressor genes, such as the *tumor protein p53* (*TP53*), *liver kinase B1* (*LKB1*), or *Kelch-like ECH-associated protein 1* (*KEAP1*) [4,5,6,7,8,9,10,11].

Poly (ADP-ribose) polymerases (PARPs) are a family of 18 protein members that function as catalytic enzymes by adding ADP-ribose polymers to lysine residues of themselves and target proteins, a process termed poly (ADP-ribosyl)ation (PARylation). Among the proteins of the PARP family, PARP-1 and -2 are abundant nuclear enzymes involved in DNA damage repair, which involves binding to DNA breaks and facilitating the localization of repair enzymes, such as breast cancer 1/2 (BRCA1/2), to the sites of DNA damage [12,13]. Olaparib, rucaparib, niraparib, and talazoparib targeting PARP1/2 have been approved by the United States Food and Drug Administration (FDA) for the treatment of breast or ovarian cancer in patients harboring HR mutations that induce synthetic lethality in the *BRCA1* or *BRCA2* gene. 

Evidence is accumulating that PARP inhibitors (PARPi) have therapeutic efficacy in cancer cells with high genomic instability by inducing synthetic lethality of cells with deficient or insufficient DNA repair [14,15,16]. Briefly, in this scenario, IR, or radiotherapy or cytotoxic chemotherapeutic agents that induce severe DNA damage could lead to insufficient DNA repair in targeted tumor cells. Consecutively, cells that adopt the “BRCAness” state could be treated with PARP inhibitor (PARPi) [17,18,19,20]. 

We have previously identified a potential novel PARPi, termed *N*-(3-(hydroxycarbamoyl)phenyl)carboxamide (designated KJ-28d). KJ-28d inhibited PARP-1/2 activities and displayed significant antitumor activity in human ovarian cancer *BRCA1*-deficient (*BRCA1* mutation at 5564G>A) SNU-251 cells [21]. In this study, we further investigated the antitumor activity of KJ-28d in *BRCA*-proficient cell lines, as well as the combination of KJ-28d and DNA damage-inducing radiotherapy or cytotoxic chemotherapeutics in human NSCLC cells. 

## 2. Results

### 2.1. KJ-28d Inhibits Growth of Human NSCLC Cells In Vitro and In Vivo

**Figure 1:**
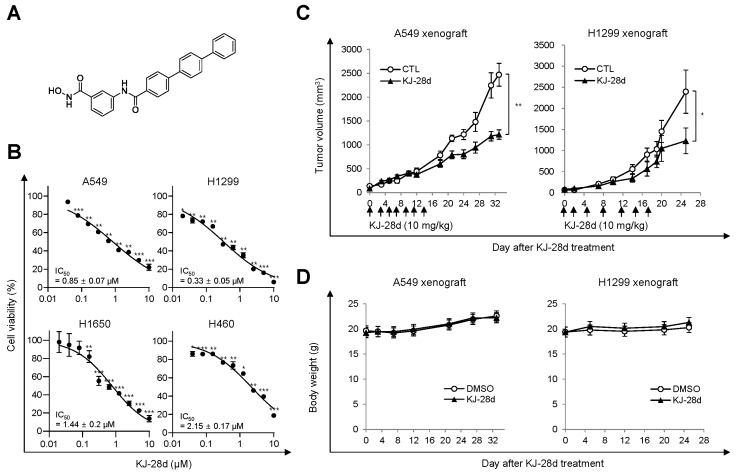
KJ-28d inhibits tumor growth of A549 and H1299 xenografts in nude mice. (**A**) The chemical structure of the KJ-28d compound. (**B**) A549, H1299, H1650, and H460 human non-small cell lung cancer (NSCLC) cells were treated with KJ-28d at the indicated concentrations for 5 days, and cell viabilities were determined by the MTT assay. Data are presented as means ± standard deviation (SD) from at least three independent experiments. * *p* < 0.05, ** *p* < 0.01, *** *p* < 0.001 versus DMSO-treated control. (**C,D**) A549 cells and H1299 cells were subcutaneously injected into the thigh of the right hind leg of BALB/c nu/nu mice (*n* = 3 per group, A549; *n* = 4 per group, H1299). Two weeks after tumor cell injection, KJ-28d (10 mg/kg) or DMSO (control) was intraperitoneally administered once every 2 or 3 days for seven times in total. (**C**) Longest (L) and shortest (W) tumor axes were measured, and tumor volume (mm^3^) was calculated as L × W^2^/2. Data shown represent average tumor volume (* *p* < 0.05, ** *p* < 0.01). Results are shown as means ± SD. (**D**) The body weights of A549 and H1299 xenograft mice were determined once a week during the experiments. Data are shown as means ± SD.

Among the reported novel PARP-1 inhibitors, we examined KJ-28d, as shown in Figure 1A, based on the prior demonstrations of its significant inhibitory activity against PARP-1, as well as antitumor activity in *BRCA*-deficient ovarian cancer cells [21]. Specifically, we assessed whether KJ-28d can inhibit the growth of *BRCA*-proficient cancer cells. Human NSCLC cells were treated with KJ-28d and growth inhibition was determined using the 3-(4,5-dimethylthiazol-2-yl)-2,5- diphenyltetrazolium bromide (MTT) assay. KJ-28d significantly inhibited the growth of A549, H1299, H460, and H1650 human NSCLC cells with a determined IC_50_ value, as shown in Figure 1B, and induced the sub-G1 phase (apoptotic cell) in A549 and H1299 cells, as shown in Supplementary Figure S1. 

We next determined whether the antitumor effect associated with the in vitro KJ-28d treatment could be translated into a similar effect in an in vivo xenograft mouse model. BALB/c-nu/nu mice were subcutaneously (*s.c.*) implanted with A549 or H1299 cells in the right hind leg, and when tumors were palpable (average diameter approximately 150 mm^3^; 10 days post-implantation), mice were intraperitoneally (*i.p.*) administered a dose of 10 mg/kg KJ-28d or DMSO (control vehicle) once every 2 or 3 days for a total of seven times. KJ-28d treatment inhibited A549 or H1299 cell-derived tumor growth by 51% and 49%, respectively, as compared with the respective vehicle, as shown in Figure 1C. Additionally, to determine the toxicity of KJ-28d, we measured the body weight of mice. Mice treated with KJ-28d did not show any difference in body weight as compared with control mice, as shown in Figure 1D. The results suggested that KJ-28d has antitumor activity for NSCLC cells in vitro and in vivo.

### 2.2. KJ-28d Induces DNA Damage and Generation of ROS in NSCLC Cells

**Figure 2:**
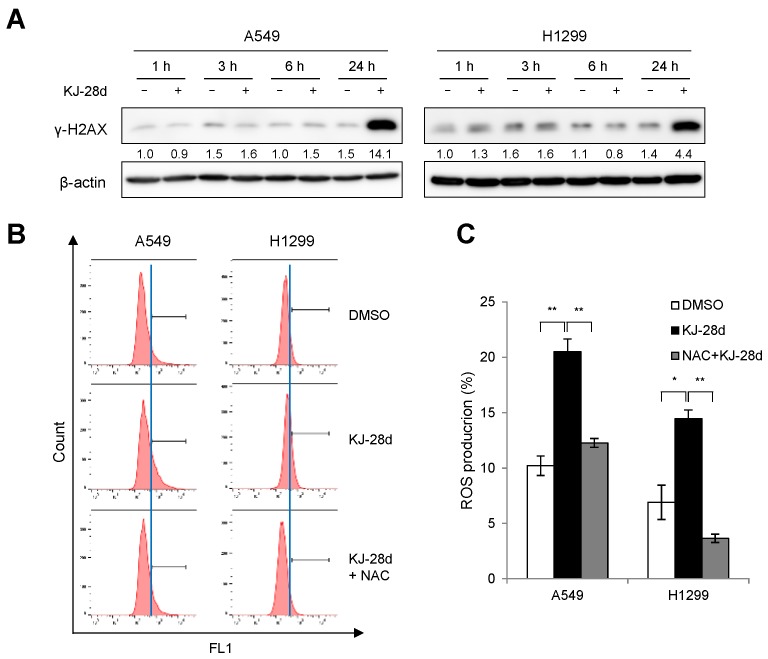
KJ-28d induces DNA damage and generation of reactive oxygen species (ROS). (**A**) A549 and H1299 cells were treated with 5 μM KJ-28d at indicated time points and immunoblotted for the detection of expression of γ-H2AX. (**B,C**) A549 and H1299 cells were treated with 5 mM NAC for 1 h, followed by KJ-28d for 24 h after incubation with 2′,7′-dichlorodihydrofluorescein diacetate (CM-H_2_DCFHDA) for 30 min. Total cellular ROS production was measured using flow cytometry. Data are representative of three independent experiments (**B**). The bar graph shows the quantitative analysis of flow cytometer data (**C**). Data are presented as the mean ± SD of three independent experiments. * *p* < 0.05, ** *p* < 0.01 versus corresponding values.

Since PARPi induce accumulation of DNA damage [22,23], we sought to determine whether KJ-28d could induce DNA damage in NSCLC cells. The DNA damage was measured in A549 and H1299 cells at different time points after KJ-28d treatment by detecting the phosphorylation on Ser139 of the H2AX (γ-H2AX) histone protein, which is an indicator of the presence of DNA double-strand damage. KJ-28d induced γ-H2AX in both cell types at the latest time point (24 h), as shown in Figure 2A. As treatment with KJ-28d resulted in inducing a DNA damage response, we also investigated whether KJ-28d could augment ROS generation in NSCLC cells. Flow cytometry analysis showed that treatment of A549 and H1299 cells with 5 μM KJ-28d led to distinctly increased ROS levels, which were then reduced following treatment with *N*-acetyl-L-cysteine (NAC), a general free radical scavenger, as shown in Figure 2B,C. These results suggested that KJ-28d is able to exhibit antitumor activities in NSCLC cells through the accumulation of DNA damage and the generation of ROS.

### 2.3. KJ-28d Potentiated Ionizing Radiation-Induced DNA Damage and Radiosensitized NSCLC Cells

**Figure 3:**
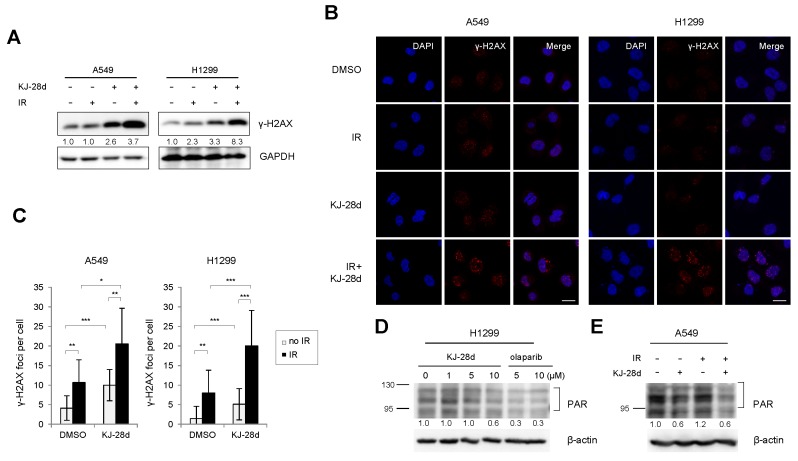
KJ-28d potentiates ionizing radiation (IR)-induced DNA damage responses. A549 and H1299 cells were treated with 5 μM KJ-28d 2 h before IR (4 Gy) and incubated for 24 h. The cell lysates were subjected to immunoblotting for detection of γ-H2AX (**A**), whereas cells were immunostained for γ-H2AX foci (red) and nuclei (DAPI: blue). Images were captured at 400× magnification. Scale bar: 20 μm (**B**). Quantification of the number of γ-H2AX foci per cell (**C**). Data represent the mean ± SD of three independent experiments. * *p* < 0.05, ** *p* < 0.01, *** *p* < 0.001 versus corresponding cells. (**D**) H1299 cells were treated with KJ-28d at indicated concentrations for 1 h. (**E**) A549 cells were treated with 5 μM KJ-28d and IR (4 Gy) and incubated for 1 h. The cell lysates were immunoblotted for the detection of expression of PAR. β-actin was used as a loading control.

As IR induces severe DNA damage, which can lead to overloading DNA repair capacity, it has been reported that PARP inhibitors enhance IR-induced DNA damage [14,17,20,22]. To examine whether KJ-28d could induce increased DNA damage in combination with IR, DNA damage was measured in A549 and H1299 cells treated with KJ-28d and IR by detecting the presence of γ-H2AX. Immunoblot analysis revealed a significant increase in the phosphorylation levels of H2AX protein as compared with KJ-28d or IR alone. Similarly, we observed high levels of staining of γ-H2AX foci in A549 and H1299 cells treated with both KJ-28d and IR, as shown in Figure 3A–C. PARylation by PARP-1 catalytic activity is a post-translational modification involved in DNA damage repair. To determine whether KJ-28d suppresses cellular PARylation, H1299 cells were treated with the indicated concentrations of either KJ-28d or olaparib, and A549 cells were treated with 5 μM KJ-28d with or without IR. We observed that 10 μM KJ-28d and 5 and 10 μM olaparib inhibited protein PARylation in H1299, as shown in Figure 3D, and 5 μM of KJ-28d inhibited IR-induced PARylation in A549 cells, as shown in Figure 3E.

**Figure 4:**
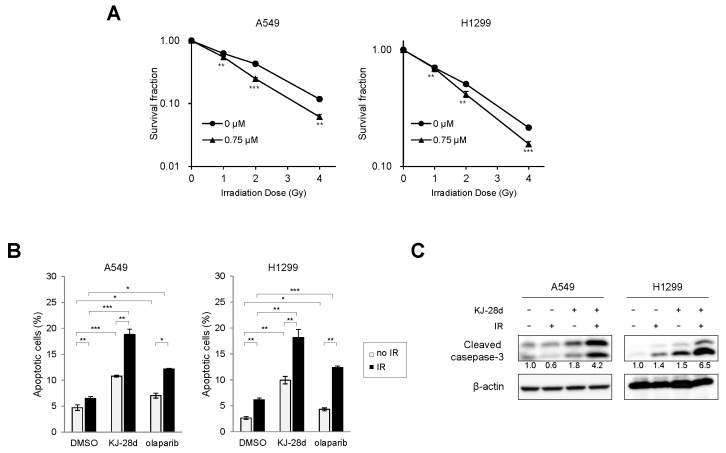
KJ-28d enhances the radiosensitivity of A549 and H1299 cells. (**A**) A549 and H1299 cells were treated with 0.75 μM KJ-28d for 2 h before IR (0, 1, 2, and 4 Gy). Clonogenic survival was measured 10 days after IR. Data are expressed as mean ± SD (*n* = 3) of the surviving fraction compared to non-irradiated cells. Colonies consisting of more than 50 cells were scored as survival colonies. (**B**) A549 and H1299 cells were treated with either 5 μM KJ-28d or 5 μM olaparib plus IR (4 Gy) for 48 h. Apoptotic cells were determined using the APC-conjugated annexin V/PI staining. Cell populations were gated into four groups, as described in Section 4. Bar graphs represent the mean percentage of early (annexin V-positive/PI-negative) and late apoptotic cells (annexin V-positive/PI-positive). Data represent the mean ± SD of three independent experiments. * *p* < 0.05, ** *p* < 0.01, *** *p* < 0.001 versus corresponding cells. (**C**) A549 and H1299 cells were treated with 5 μM KJ-28d plus IR (4 Gy), and the cell lysates were subjected to immunoblotting for detection of cleaved caspase-3. β-actin was used as a loading control.

Since KJ-28d potentiated IR-induced DNA damage in NSCLC cells, we further examined whether KJ-28d inhibited IR-induced cell growth. A549 and H1299 cells were treated with KJ-28d 2 h before IR. The clonogenic survival assay revealed that KJ-28d radiosensitized both cell lines, as shown in Figure 4A. Dose enhancement ratios (DER) of 0.75 μM KJ-28d-treated (at a surviving fraction of 0.37) to DMSO-treated cells were 1.5 and 1.23 in A549 and H1299 cells, respectively. We next determined whether treatment with KJ-28d could induce apoptotic cell death in A549 and H1299 cells. Apoptotic cell populations of these cell lines were detected using flow cytometer analysis with annexin V/propidium iodide (PI) staining. Following treatment with 5 μM of KJ-28d and IR, the number of A549 and H1299 cells undergoing both early-stage (annexin V-positive/PI-negative) and late-stage (annexin V-positive/PI-positive) apoptosis increased significantly by 1.5-fold compared to KJ-28d alone, respectively, as shown in Figure 4B. In addition, KJ-28d plus IR treatment increased the cleavage of caspase-3 in both cell lines, as shown in Figure 4C. Taken together, these results indicated that KJ-28d enhanced both IR-induced DNA damage and apoptotic cell death in A549 and H1299 human NSCLC cells.

### 2.4. The Combination of KJ-28d and DNA Damage-Inducing Chemotherapeutic Agents Synergistically Inhibits NSCLC Cell Growth

**Figure 5:**
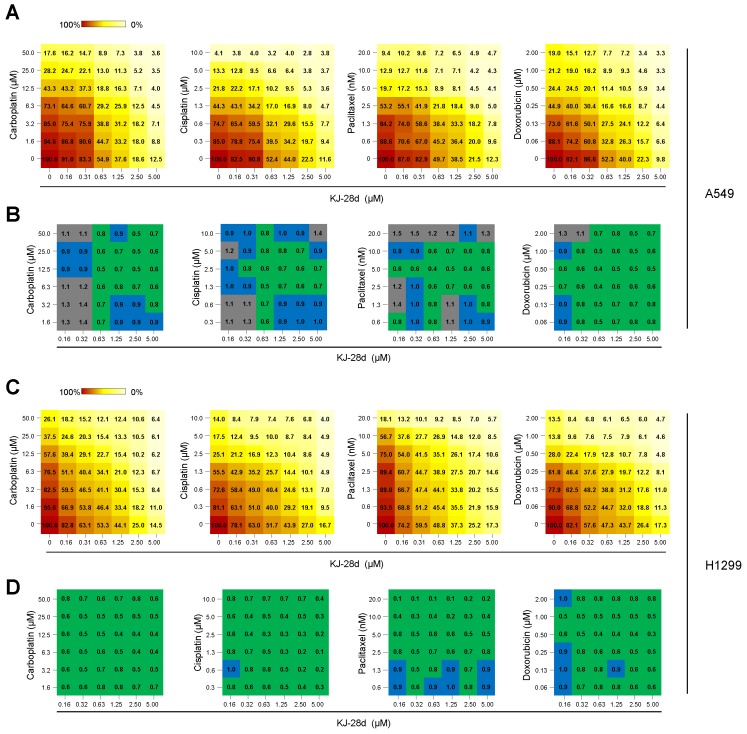
The combination of KJ-28d and DNA damage-inducing chemotherapeutic agents synergistically inhibits growth of A549 and H1299 cells. A549 (**A**) and H1299 (**C**) cells were treated with the single or combined administration of KJ-28d and carboplatin, cisplatin, paclitaxel, or doxorubicin at indicated concentrations. Cell viability was determined 5 days after the treatment by MTT assay. Relative viability (normalized to DMSO-treated cells) is shown for each combination at indicated concentrations. Data are from one representative experiment of three independently repeated experiments. (**B,D**) Summary of tables showing combination index (CI) scores of KJ-28d and each chemotherapeutic drug combined at indicated concentrations in A549 (**B**) and H1299 cells (**D**). CI scores were calculated using the CompuSyn software and categorized as synergistic (CI < 0.9, green), additive (1.1 > CI ≥ 0.9, blue), or antagonistic (CI ≥ 1.1, gray). Each CI score was one representative datum from treatment with the indicated concentrations of single- and paired compounds from more than three independent experiments.

In preclinical and clinical studies of advanced NSCLC treatment, administration of PARPi in combination with DNA-damaging therapeutic agents, such as platinum-based compounds, taxane-based compounds, and topoisomerase inhibitors, has demonstrated enhanced cytotoxicity [24,25,26]. As KJ-28d enhanced radiosensitivity of A549 and H1299 cells, we expected that KJ-28d could increase cytotoxicity when combined with treatment with DNA DSB-inducing agents. A549 and H1299 cells were treated with KJ-28d and carboplatin, cisplatin, paclitaxel, or doxorubicin and evaluation of the synergistic effect of each pair-compound on cell viability was assessed using the MTT assay. Compared to treatment with KJ-28d or each compound alone, combination treatments showed a strong synergistic effect in both A549 and H1299 cells, decreasing cell viability in a dose-dependent manner, as shown in Figure 5A,C. To interpret the effects of all drug combinations, cell viability observed with each concentration of a KJ-28d-compound pair was converted to a combination index (CI) score using the CompuSyn software by Chou–Talalay [27]. CI scores were categorized as synergistic (CI < 0.9, green), additive (1.1 > CI ≥ 0.9, blue), or antagonistic (CI > 1.1, gray). We observed synergistic growth inhibition with a wide range of concentrations of KJ-28d and DSB-inducing agents in both cell lines, as shown in Figure 5B,D. Taken together, these results supported the conclusion that KJ-28d enhances the sensitivity of NSCLC cells to IR or chemotherapeutic agents of DNA-induced damage.

## 3. Discussion

We previously identified KJ-28d as a novel PARP inhibitor that leads to increased cytotoxicity in human ovarian cancer *BRCA-1*-deficient SNU-251 (*BRCA1* mutation at 5564G>A) cells, as well as in triple-negative human ovarian cancer *BRCA1* heterozygous (*BRCA1*+/−) MDA-MB-231 cells [21]. In this study, we further investigated the antitumor activity of KJ-28d on *BRCA*-proficient NSCLC cancer cells, as well as a combination approach with DNA damage-inducing agents to evaluate the synergistic therapeutic efficacy in human NSCLC cells.

NSCLC is a type of cancer with a high mortality rate. Most patients with NSCLC receive treatment with platinum-based drugs, such as cisplatin or carboplatin, as first-line standard therapy. However, many patients who initially benefit from such chemotherapies gradually acquire chemoresistance. Thus, the development of novel therapies and strategies, including combination therapies, is needed. KJ-28d can inhibit the growth of various cancer cells, including NSCLC, breast, and colorectal cancer cells, as shown in Supplementary Figure S2. We specifically focused on NSCLC and revealed its antitumor effects on A549 and H1299 cells both in vitro and in vivo. A549 cells have a *KRAS* oncogenic mutation with c-Myc amplification, while H1299 cells are *TP53* mutant cells, as shown in Supplementary Table S1. Many studies have shown that KRAS-driven c-Myc amplification and TP53 correlate with genomic instability, thereby compromising DNA damage repair (DDR), causing the cells to be vulnerable to DDR inhibiting agents [28]. We observed that KJ-28d induced DNA damage and ROS generation in A549 and H1299 cells, which may facilitate cell death of NSCLC cells. The suppressed cellular PARylation might contribute to inducing DNA damage responses. Recent studies have suggested that PARPi can induce ROS generation due to DNA double stranded breaks. This is considered a characteristic of PARP inhibitors in DNA repair-deficient cells, as well as tumor suppressors or oncogene-mutated cells. Thus, PARPi may crosstalk with other signals [20,29]. In that regard, FDA-approved PARPi compounds have been studied to expand clinical use of PARPi in NSCLC [26,30,31].

Although the rationale for the use of PARPi was based on the reported induced synthetic lethality shown in *BRCA*-deficient cancer cells, preclinical and clinical studies suggested that PARPi could potentially be used as a combination partner with DNA-damaging agents in *BRCA*-proficient cancer cells [26,32,33]. Approximately 50% of patients with NSCLC receive radiotherapy during their treatment course [34]. Radiotherapy is an effective treatment modality, causing severe DNA damage. However, it can only treat tumors at defined doses because of the side effects to surrounding normal tissues. Although PARPi is not yet considered as a radiosensitizer in patients with ovarian cancer harboring *BRCA1/2* mutations in the clinic, the combination of radiotherapy with PARPi could provide promising synergistic therapeutic effects. For that reason, it has been studied in human NSCLC xenografts [35,36,37]. Olaparib is the first PARPi approved for the treatment of refractory ovarian cancer harboring *BRCA1* or *BRCA2* mutations. We have previously shown that treatment with KJ-28d induced more frequent apoptotic cell death in *BRCA* mutated ovarian cancer cells than olaparib did. It was noteworthy in this study that the combination of KJ-28d and IR also induced significantly more apoptotic cell deaths in NSCLC cells compared to combination treatment with olaparib and IR. Since we initially identified KJ-28d as a PARPi, further studies need to be conducted to elucidate other modes of action, as they may be cytotoxic. Platinum-based agents are widely used for a broad range of solid tumors, including NSCLC, and are the most commonly studied combination partners of PARPi [26,31,38]. Likewise, our results demonstrated that KJ-28d significantly enhanced the sensitivity of A549 and H1299 cells to carboplatin or cisplatin. In addition, we observed synergistic cell growth inhibitions of both cell populations when treated with a combination of KJ-28d with either paclitaxel or doxorubicin that constitute strong DNA-damaging therapeutic agents.

Besides the demonstrated inhibitory PARP activity of KJ-28d, the mechanisms responsible for KJ-28d-induced cytotoxicity in wild-type *BRCA* cancer cells have not been fully uncovered in this study. We suggest that KJ-28d might be involved in the antitumor activity exhibited in NSCLC cells with wild-type *BRCA* based on the experimentally exhibited markedly induced DNA DSBs and ROS generation at the latest examined time point (24 h). Structurally, KJ-28d contains the hydroxamic acid moiety found in histone deacetylase inhibitors (HDACi), including suberoylanilide hydroxamic acid. The hydroxamic acid moiety of HDACi acts as a chelator for zinc ions in the active site of histone deacetylases. To elucidate the possibility of HDAC inhibition, we examined the inhibitory activities of 1–11 HDAC isoforms using in vitro enzyme assays but observed little inhibitory activities of HDACs at KJ-28d concentrations under 5 μM, as shown in Supplementary Table S2. Further structure–activity relationship studies are required to understand the inhibitory activities of PARP-1/2 as a PARPi and to investigate the mechanisms associated with KJ-28d-induced growth inhibition and apoptotic cell death in NSCLC cells.

In summary, KJ-28d was cytotoxic to *BRCA*-proficient cancer cells, including NSCLC cells. Treatment with KJ-28d before IR led to increased DNA damage responses compared to treatment with KJ-28d or IR alone, resulting in the induction of apoptotic cell death. The combination of KJ-28d with carboplatin, cisplatin, paclitaxel, or doxorubicin considerably inhibited cell proliferation. In this context, KJ-28d might act as an effective anti-cancer therapeutic agent against both *BRCA*-deficient and -proficient cancer cells and might have further potential as an adjuvant when used in combination with radiotherapy or DNA-damaging agents. However, further investigations into the mechanisms of action of KJ-28d in cancer cells are warranted.

## 4. Materials and Methods

### 4.1. Cell Culture

Human lung cancer cells (A549, H1299, H460, and H1650) were obtained from American Type Culture Collection (ATCC, Manassas, VA, USA). Cells were maintained in Roswell Park Memorial Institute (RPMI) 1640 medium (Welgene, Gyeongsangbuk-do, Korea) supplemented with 10% fetal bovine serum (Welgene) and 100 units/mL penicillin–streptomycin solution (Gibco, Grand Island, NY, USA) at 37 °C in a humidified 5% CO_2_ atmosphere.

### 4.2. Reagents

The PARPi olaparib (AZD2281) was purchased from Selleckchem (Houston, TX, USA). Doxorubicin was purchased from Sigma-Aldrich (St. Louis, MO, USA). All reagents, including carboplatin (Dong-A ST, Seoul, Korea), cisplatin (JW Pharmaceutical, Seoul, Korea), and paclitaxel (Samyang Biopharm, Gyeonggi-do, Korea) were dissolved in dimethyl sulfoxide (DMSO; Sigma-Aldrich). MTT was purchased from Amresco (Solon, OH, USA). Primary antibodies used in this study included the following; anti-cleaved caspase 3 (D175, #9661; Cell Signaling Technology; Danvers, MA, USA), phospho-histone H2AX (Ser 139, sc-517348), and glyceraldehyde 3-phosphate dehydrogenase (GAPDH, sc-47724; Santa Cruz Biotechnology; Dallas, TX, USA), and anti-β-actin (A5441; Sigma-Aldrich) antibodies.

### 4.3. Cell Viability Assay

Cell viability was assessed using the MTT colorimetric assay. Cells (6 ~ 8 × 10^2^ cells/well) were seeded into 96-well plates and treated with various concentrations of KJ-28d or a combination of anti-cancer drugs or IR. After 5 days of treatment, 10 μL MTT (0.5 mg/mL) was added, and cells were further incubated for 3 h. After removal of the supernatant, the resultant pellet was dissolved in DMSO. The absorbance of the resultant formazan was measured at 540 nm using a Multiskan EX plate reader (Thermo LabSystems, Waltham, MA, USA).

### 4.4. Tumor Xenograft Mouse Models

A549 and H1299 human lung cancer cell xenografts were established by *s.c.* implantation of 2 × 10^6^ cultured cells into the thigh of the right hind leg of six-week-old mice. When tumor volumes had reached approximately 100 mm^3^, KJ-28d (10 mg/kg) was administered *i.p.* once per 2 or 3 days for seven times in total. All animal experiments were reviewed and approved by the Institutional Animal Care and Use Committee of Korea Institute of Radiological and Medical Sciences (kirams2018-0063, 2018).

### 4.5. Detection of Intracellular ROS

Either DMSO- or KJ-28d-treated cells (5 × 10^5^) were further treated with 10 μM CM-H_2_DCFH-DA (Thermo Fisher Scientific, Rockford, IL, USA) for 30 min and then washed with phosphate-buffered saline (PBS). After trypsinization, cells were collected, washed, and resuspended in PBS. Inhibition of ROS was evaluated by treating cells with 5 mM NAC 2 h prior to KJ-28d treatment. Intracellular ROS levels were detected using a CyFlow cube 6 flow cytometer (Sysmex Partec, Gorlitz, Germany) at excitation/emission wavelengths of 488/525 nm.

### 4.6. Immunoblot Analysis

A549 and H1299 cells (1 × 10^6^) were seeded onto a 60 mm dish. Cell lysates were prepared by extracting proteins with radioimmunoprecipitation assay (RIPA) lysis buffer (Millipore, Billerica, MA, USA) supplemented with a protease inhibitor cocktail (Thermo Fisher Scientific). Equal amounts of proteins were separated using SDS-PAGE on 8%–13% gels and transferred to nitrocellulose membranes (Bio-Rad, Hercules, CA, USA). Membranes were blocked with 5% skim milk in Tris-buffered saline-Tween 20 (TBST; 150 mM NaCl, 10 mM Tris, 0.2% Tween 20; Sigma-Aldrich), followed by overnight incubation with primary antibodies at 4 °C. Blots were developed using peroxidase-conjugated secondary antibody, and immunoreactive proteins were visualized using enhanced chemiluminescence reagents, according to the manufacturer’s recommendations (Amersham, GE Healthcare, Buckinghamshire, UK). Protein bands were visualized using an ImageQuant LAS 4000 mini digital imaging system (GE Healthcare). Protein levels were analyzed using Image J software (National Institutes of Health, Bethesda, MD, USA). Experiments were repeated at least three times.

### 4.7. Clonogenic Assay

Cells were seeded on 60 mm culture dishes at various densities and then treated with DMSO or KJ-28d (0.75 μM). After 2 h, cells were treated with the indicated doses of 137Cs γ-radiation. After 10 d, colonies were fixed and stained with 1.5% methylene blue (Sigma-Aldrich) in methanol solution. Colonies containing >50 cells were counted. DER was calculated as the dose (Gy) of radiation that yielded a surviving fraction of 0.1 for DMSO-treated cells divided by that dose for KJ-28d-treated cells. The experiment was performed in triplicate.

### 4.8. Annexin V/PI-Based Flow Cytometric Analysis

Annexin V assays were performed according to the manufacturer’s protocol (BD Pharmingen, San Diego, CA, USA). Briefly, 10,000 cells were plated into 60 mm plates and treated with KJ-28d (10 μM) for 48 h. Cells were harvested and incubated with 4 μL allophycocyanin (APC)-conjugated annexin V (20 μg/mL) and 4 μL PI (50 μg/mL) for 15 min. Fluorescence analyses were performed using flow cytometry (CyFlow cube 6). Cells were classified as early apoptotic (annexin V-positive/PI-negative), late apoptotic/necrotic (annexin V-positive/PI-positive), necrotic/dead (annexin V-negative/PI-positive), and live (annexin V-negative/PI-negative). Flow cytometry data were analyzed using FlowJo software (TreeStar Inc., Ashland, OR, USA).

### 4.9. Combination Index

CI scores were calculated using the CompuSyn software by Chou (CompuSyn Inc., Paramus, NJ, USA) [27] based on cell viability after treatment with single and paired drug concentrations. The CI equation for two drugs used is given below:

$$\mathrm{CI}\;=\frac{\left(D\right)A}{\left(\mathrm{Dx}\right)A}+\frac{\left(D\right)B}{\left(\mathrm{Dx}\right)B}$$

where (Dx)A is the concentration of drug A alone that inhibits x%, (Dx)B is the concentration of drug B alone that inhibits x%, (D)A or (D)B is the portion of drug A or drug B in the combination (D)A + (D)B that inhibits x%. Thus, (D)A + (D)B also inhibits x%.

### 4.10. Statistical Analyses

Results are shown as means ± SD. Data were analyzed using the two-tailed Student’s *t*-test. Analysis of variance (ANOVA) and Tukey’s post hoc test were used for 2 or 3 group comparisons. Differences between groups with *p* values < 0.05 were considered statistically significant.

## Supplementary Materials

Supplementary materials can be found at https://www.mdpi.com/1422-0067/20/23/6026/s1.

## Abbreviations

PARP-1	Poly (ADP-ribose) polymerase-1
PARPi	PARP inhibitor(s)
PARylation	Poly ADP-ribosylation
NSCLC	non-small cell lung cancer
DSB	double-strand breaks
IR	ionizing radiation
ROS	reactive oxygen species
CI	combination index
