# Supplementary material for: A Small Compound KJ-28d Enhances the Sensitivity of Non-Small Cell Lung Cancer to Radio- and Chemotherapy
Source: Int J Mol Sci. 2019 Nov 29;20(23):6026. doi: 10.3390/ijms20236026 (PMC6928747; doi:10.3390/ijms20236026)
Supplement: Supplementary file 1 [file ijms-20-06026-s001.pdf]

# Supplementary Materials

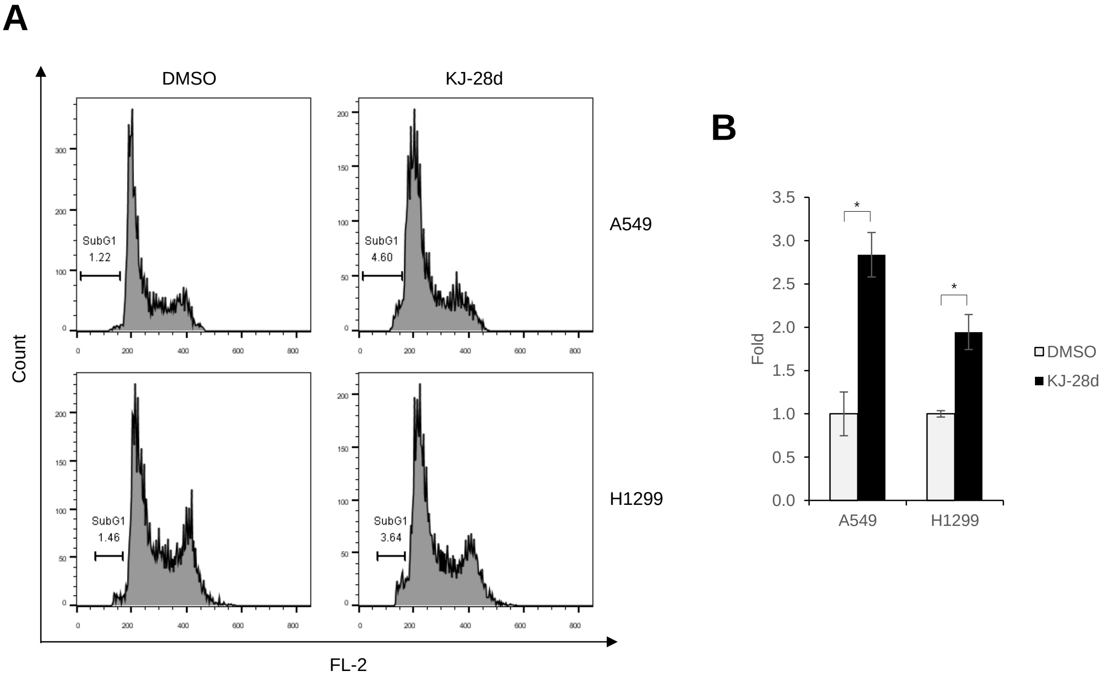


**Figure S1.** KJ-28d induces sub-G1 phase in A549 and H1299 cells. Human NSCLC A549 and H1299 cells were treated with 5 μM KJ-28d for 24 h and stained with PI. The cell cycle distribution was analyzed by flow cytometry (**A**). The bar graph shows the quantitative analysis of FACS data (**B**). *$p$ < 0.05 versus corresponding values.

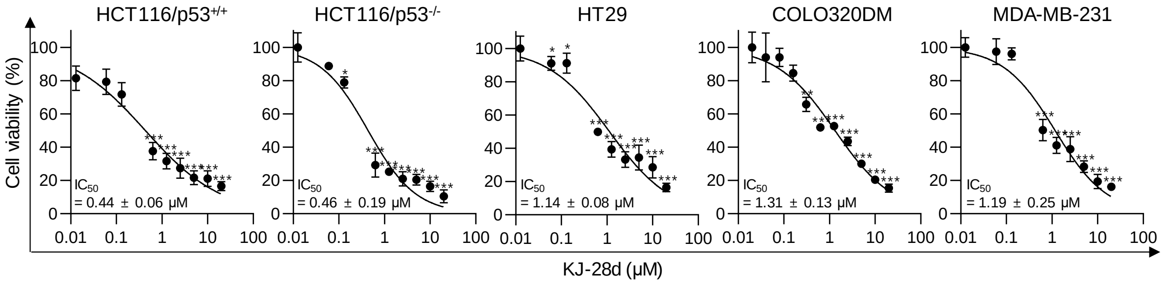


**Figure 2.** KJ-28d inhibits the proliferation of human cancer cells. Human colorectal cancer $p53^{+/+}$ HCT116, $p53^{-/-}$ HT29, and COLO320DM cells and human breast cancer MDA-MB-231 cells were treated with KJ-28d at indicated concentrations for 5 days and cell viabilities were determined using the MTT assay. Data are presented as means ± standard deviation (SD) from at least three independent experiments. *$p$ < 0.05, **$p$ < 0.01, ***$p$ < 0.001 versus DMSO-treated control. .

**Table 1.** NSCLC cell lines used in this study.

| Cell line | KRAS | EGFR | TP53 | KEAP1 | Remarks |
|---|---|---|---|---|---|
| **A549** | MT (missense) 34G>A, G12S | WT | WT | MT (missense) 997G>T, G333C | c-MYC: amplified |
| **H1299** | WT | WT | Null | WT | - |
| **H1650** | WT | MT (deletion) c2235-2249del, E746-A750del | c.673-2A>G | WT | TP53: deletion in intron |
| **H460** | MT (missense) 183A>T, Q61H | WT | WT | MT (missense) 706 G>C, D236H | c-MYC: amplified |

Note: Mutation status of each cell line was determined from the Sanger Catalogue of Somatic Mutations in Cancer database (COSMIC, http://www.sanger.ac.uk/genetics/CGP/cosmic). Abbreviation: WT, wild-type; MT, mutation

**Table S2**. Activities (%) of KJ-28d (5 μM) against HDAC isoenzymes *in vitro.*

| HDAC isoenzyme | Activity (%) |
|---|---|
| DMSO | 100.0 |
| HDAC1 | 82.4 |
| HDAC2 | 100.0 |
| HDAC3 | 100.0 |
| HDAC4 | 98.4 |
| HDAC5 | 92.5 |
| HDAC6 | 78.3 |
| HDAC7 | 100.0 |
| HDAC8 | 83.2 |
| HDAC9 | 100.0 |
| HDAC10 | 100.0 |
| HDAC11 | 78.3 |

## Supplementary Materials and Methods

*Sub-G1 analysis.* Cells were treated with 5 μM KJ-28d. After 24 h treatment, cells were fixed in 70% cold ethanol overnight. For cell cycle analysis, fixed cells were treated with RNase for 20 min before addition of 5 μg/mL PI and analyzed by flow cytometry (CyFlow cube 6).

*Cell culture.* Human breast cancer cells (MDA-MB-231) and human colon cancer cells (HT29 and COLO320-DM) were obtained from ATCC (American Type Culture Collection). $p53^{+/+}$ HCT116 and $p53^{-/-}$ HCT116 cells were kindly provided by Dr. Bert Vogelstein (Johns Hopkins University, Baltimore, MD, USA). Cells were maintained in Roswell Park Memorial Institute (RPMI) 1640 medium (Welgene) supplemented with 10 % fetal bovine serum (FBS; Welgene) and 100 units/mL penicillin streptomycin solution (Gibco) at 37 °C in a humidified 5 % $CO_2$ atmosphere.

*In vitro enzyme assay.* Enzyme activities of KJ-28d against HDAC isoenzymes (1~11) were performed Reaction Biology Corp. (Malvern, PA, USA).
